# Lateral interbody release for fused vertebrae via transpsoas approach in adult spinal deformity surgery: a preliminary report of radiographic and clinical outcomes

**DOI:** 10.1186/s12891-022-05204-0

**Published:** 2022-03-14

**Authors:** Masanari Takami, Shunji Tsutsui, Yasutsugu Yukawa, Hiroshi Hashizume, Akihito Minamide, Hiroshi Iwasaki, Keiji Nagata, Ryo Taiji, Andrew J. Schoenfeld, Andrew K. Simpson, Hiroshi Yamada

**Affiliations:** 1 Department of Orthopaedic Surgery, Wakayama Medical University, 811-1 Kimiidera, Wakayama, 641-8510 Japan; 2 Department of Orthopaedic Surgery, Brigham and Women’s Hospital, Harvard Medical School, 75 Francis Street, Boston, MA 02115 USA

**Keywords:** Lateral interbody release technique, Lateral lumbar interbody fusion, Fused vertebrae, Anterior column realignment, Adult spinal deformity, Corrective fusion surgery, Degenerative lumbar kyphoscoliosis

## Abstract

**Background:**

Lateral interbody release (LIR) via a transpsoas lateral approach is a surgical strategy to address degenerative lumbar scoliosis (DLS) patients with anterior autofusion of vertebral segments. This study aimed to characterize the clinical and radiographic outcomes of this lumbar reconstruction strategy using LIR to achieve anterior column correction.

**Methods:**

Data for 21 fused vertebrae in 17 consecutive patients who underwent LIR between January 2014 and March 2020 were reviewed. Demographic and intraoperative data were recorded. Radiographic parameters were assessed preoperatively and at final follow-up, including segmental lordotic angle (SLA), segmental coronal angle (SCA), bone union rate, pelvic incidence (PI), lumbar lordosis (LL), pelvic tilt, sacral slope, PI-LL mismatch, sagittal vertical axis, Cobb angle, and deviation of the C7 plumb line from the central sacral vertical line. Clinical outcomes were evaluated using Oswestry Disability Index (ODI), visual analog scale (VAS) scores for low back and leg pain, and the short form 36 health survey questionnaire (SF-36) postoperatively and at final follow-up. Complications were also assessed.

**Results:**

Mean patient age was 70.3 ± 4.8 years and all patients were female. Average follow-up period was 28.4 ± 15.3 months. Average procedural time to perform LIR was 21.3 ± 9.7 min and was not significantly different from traditional lateral interbody fusion at other levels. Blood loss per single segment during LIR was 38.7 ± 53.2 mL. Fusion rate was 100.0% in this cohort. SLA improved significantly from − 7.6 ± 9.2 degrees preoperatively to 7.0 ± 8.8 degrees at final observation and SCA improved significantly from 19.1 ± 7.8 degrees preoperatively to 8.7 ± 5.9 degrees at final observation (*P* < 0.0001, and < 0.0001, respectively). All spinopelvic and coronal parameters, as well as ODI and VAS, improved significantly. Incidence of peri- and postoperative complications such as iliopsoas muscle weakness and leg numbness in patients who underwent LIR was as much as XLIF. Incidence of postoperative mechanical failure following LIR was also similar to XLIF. Reoperation rate was 11.8%. However, there were no reoperations associated with LIR segments.

**Conclusions:**

The LIR technique for anterior column realignment of fused vertebrae in the context of severe ASD may be an option of a safe and effective surgical strategy.

## Background

Enthusiasm for corrective surgery for adult spinal deformity (ASD) has increased over the last 20 years, fueled by recognition of the importance of sagittal and coronal spinal alignment on health-related quality of life and growth of procedural options to achieve these goals [1–3]. Procedural strategies for deformity correction have shifted over time from focal three column osteotomies to increased utilization of multilevel interbody fusions [4]. These multilevel interbody corrections achieve more gradual segmental correction while mitigating the higher risk profiles associated with pedicle subtraction osteotomies (PSO) and vertebral column resections (VCR) [5–12].

Lateral lumbar interbody fusions (LLIF) have evolved from more limited degenerative operations to a tool for deformity correction via multi-segmental interbody approaches [4, 13–20]. LLIF has several technique-specific advantages, including greater capacity for segmental coronal correction and high fusion rates, due to the ability to release osteo-ligamentous structures and large cage footprint [16–20]. Additionally, these procedures have demonstrated decreased intraoperative blood loss as compared to three column osteotomy corrections [13–15].

More severe degenerative scoliosis cases can exhibit autofusion of multiple vertebral segments. In many of these cases, there is partial fusion of the vertebral bodies with some area of remnant disc space. Currently, such clinical realities are felt to obviate traditional interbody fusion techniques and surgeons typically rely on three column osteotomies or correction through other vertebral segments. If osteotomy of the fused portion of the vertebral segment was achievable via a lateral transpsoas approach and interbody fusion in these cases, this might allow surgeons to avoid three column osteotomies and the higher morbidity associated with these procedures.

Given the expanding role of multi-segment lateral interbody fusion for deformity correction, and the paucity of evidence for lateral-based techniques to address vertebral autofusion in more severe deformity, we sought to develop a lateral interbody release (LIR) technique for addressing these cases.

To the best of our knowledge, release for fused vertebrae using LLIF technique has not been previously explored in the literature. In this study we aimed to characterize the radiographic and clinical outcomes of a lumbar reconstruction strategy including lateral interbody release to achieve anterior column realignment in patients with ASD.

## Methods

This retrospective review was approved by the appropriate institutional review board prior to initiation. This study met guidelines of the responsible local governing agency and complied with the principles of the Declaration of Helsinki. The patients or their families were informed that the data from the cases would be submitted for publication, and their written consent was obtained.

### Materials

This retrospective cohort study includes 17 consecutive patients (21 fused vertebral levels) with degenerative lumbar kyphoscoliosis or kyphosis, who underwent the LIR procedure as part of their ASD surgery between January 2014 and March 2020. All included individuals had completed at least 1-year follow-up following the procedure.

### Surgical procedure

**Fig. 1:**
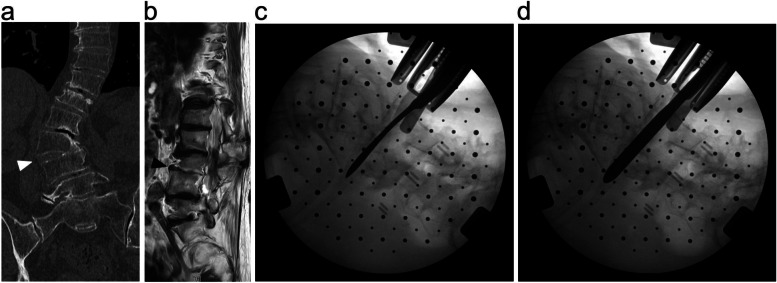
**a** This degenerative lumbar kyphoscoliosis case had fused vertebrae at L3–4 (white arrowhead). Anterior vertebral autofusion was found in the concave side of the lumbar curve. **b** In the same case, the remnant disc was found on T2 weighted image of magnetic resonance image at the intervertebral space of L3/4 level (black arrowhead). **c** A Cobb elevator with 18 mm width was inserted into the disc space from the non-fused side and penetrated to the contralateral side under a fluoroscope. **d** Subsequently, a 6 mm thick Paddle Sizer® was inserted to completely accomplish the release between the fused vertebrae

LIR was performed on fused vertebrae resulting from advanced degenerative changes in the lumbar spine (Fig. 1a). All patients undergo pre-operative scoliosis series, computed tomography (CT) with multiplanar reconstruction and MRI imaging. We performed LIR when there was radiographic evidence of fused vertebrae but low signal intensity in the interbody space was observed on T2 weighted magnetic resonance imaging (MRI), indicating a remnant of the disc space (Fig. 1b). We would not perform LIR if vital structures such as the aorta and the vena cava were appreciated to be in the path of the interbody approach on preoperative radiological images. The approach side was determined by whether interbody access was feasible at the L4/5 level. In addition, the LIR approach is easier from the non-fused side of fused vertebrae.

LIR was performed when it was necessary to release the fused vertebrae in order to obtain the target lumbar lordosis (LL) determined using the Scoliosis Research Society-Schwab criteria [2]. Patient positioning follows the standard technique for transpsoas interbody approaches, as does the initial surgical access which employs the standard retractor (Maxcess®, NuVasive, San Diego, CA, USA). Once the operative level has been accessed, a Cobb elevator with 18 mm width was inserted into the disc space from non-fused side. The Cobb elevator was penetrated to the contralateral side of the fused vertebrae under a fluoroscope (OEC 9900 series, GE Health Care Japan co., Hibi, Tokyo) (Fig. 1c). A straight chisel with 7 mm width was sometimes used when there was resistance to advance the Cobb elevator. Mobility between the fused vertebrae may be appreciated by gently rotating the Cobb elevator at this time. This will also confirm that a release has been achieved.

Next, a 6 mm thick Paddle Sizer® (NuVasive, San Diego, CA, USA) was inserted to completely accomplish the release between the fused vertebrae (Fig. 1d). This is followed by the insertion of a Cobb elevator and Paddle Sizer with 18 mm width in sequence to complete release. After inserting trial devices, the cage with an anteroposterior width of 18 mm was inserted according to the usual extreme lateral interbody fusion (XLIF) procedure. At this time, the cage was inserted so as to partially rest on the released osteophyte or the osteosclerotic end plate on the convex side of the curvature. Autologous iliac crest bone and artificial bone made of hybridized hydroxyapatite and type I collagen (ReFit®, HOYA Technosurgical Co., Tokyo, Japan) were mixed (50/50 proportion) and inserted into the cage.

After the anterior procedure including LIR was completed, the patient was placed in the prone position and the posterior corrective fusion surgery was performed as a single staged surgery. The posterior fusion was typically performed from the thoracic spine to the pelvis including L5/S1 posterior lumbar interbody fusion (PLIF). If there was poor flexibility of the spinal motion, grade 1 or 2 osteotomy, as suggested by the Scoliosis Research Society-Schwab criteria [21], was also performed. Spinal kyphosis was corrected by using the cantilever technique with bilateral S1 pedicle screws and bilateral S2 alar iliac screws. Two bent titanium rods with 5.5 mm diameter were connected to the pedicle screws. If the target LL was achieved after the anterior procedure and there were no severe atrophy of the back muscles and no severe degenerative changes of the cranial and caudal discs adjacent to the range of the anticipated fusion construct, short fusion within the lumbar spine was selected. The patient was mobilized as soon as possible following the surgery and wore a hard corset for 3 months postoperatively.

### Patient demographic, clinical, and surgical data

**Fig. 2:**
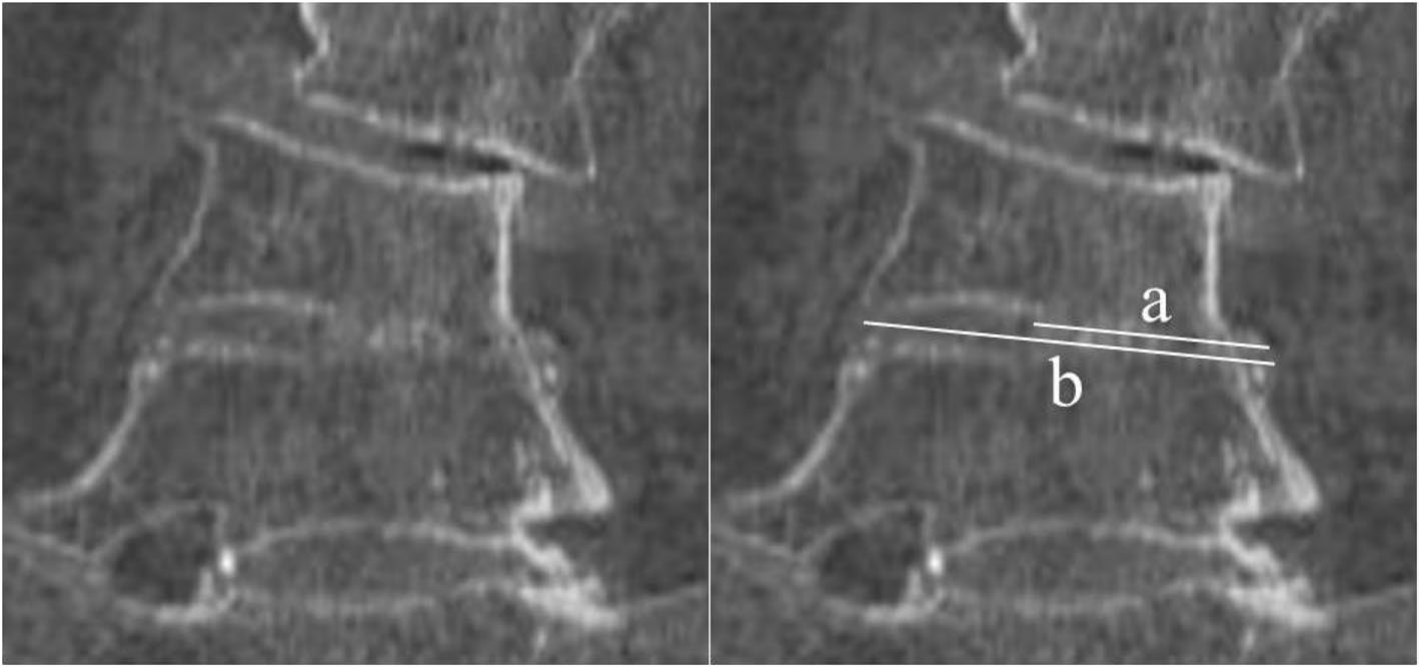
The fusion ratio is preoperatively calculated by dividing the length of the ossified portion of the intervertebral space (**a**) by the total length of the intervertebral space (**b**) on the exact coronal plane image using multiplanar reconstruction of computed tomography

Patient charts were abstracted to obtain baseline demographic and surgical characteristics of include patients. These consisted of biologic sex, age, body mass index (BMI), bone density (T-score), injection of teriparatide 2 months or more before surgery, follow-up periods, and treated intervertebral levels, surgical access side (convex or concave) and range of fusion levels. Preoperative fusion ratio (FR) of auto-fused vertebrae was assessed according to the ratio of the length of the autofused portion to the total height of the intervertebral space on coronal plane imaging using multiplanar reconstruction (MPR) of the CT scan (Fig. 2).

### Evaluation of postoperative LIR segments

The time from installation to removal of the Maxcess® retractor was measured at 17 fused vertebrae treated with LIR and 28 intervertebral segments operated with usual XLIF in the non-fused interbody. An amount of blood loss in the anterior surgery was investigated from an operation record and an amount of blood loss per single segment was calculated.

The segment operated with LIR was evaluated radiologically. Segmental lordotic angle (SLA, the angle measured between the superior endplate of the fused vertebra cranially and inferior endplate of the fused vertebra caudally in the sagittal plane) and segmental coronal angle (SCA, the angle measured between the superior endplate of the fused vertebra cranially and inferior endplate of the fused vertebra caudally in the coronal plane) was measured preoperatively and at the final observation. SLA was measured by creating an exact sagittal image of the center of the vertebral body using MPR of CT. SCA was similarly measured by creating an exact coronal image of the center of the vertebral body. Bone union was investigated using CT 1-year after surgery. Patterns of bone union were assessed in the fused segments treated by LIR according to the Proietti classification [17].

To control for intraobserver variability, the measurements of SLA and SCA in all 21 fused vertebrae were evaluated by the same observer, > 4 weeks after the first reading. The measurements were also evaluated by two spine surgeon supervisors certified by the Japanese Society for Spine Surgery and Related Research to determine interobserver variability.

### Evaluation of global spine alignment

For the spinopelvic parameter, pelvic incidence (PI), lumbar lordosis (LL), pelvic tilt (PT), sacral slope (SS), PI-LL mismatch, and sagittal vertical axis (SVA), and as coronal alignment parameters Cobb angle (the angle measured between the superior endplate of the most tilted vertebra cranially and the inferior endplate of the most tilted vertebra caudally) and C7-CSVL (deviation of the C7 plumb line from the central sacral vertical line) were evaluated in 14 patients (82.4%) with minimum 2-years follow-up before surgery and at the final observation. All spinopelvic and coronal parameters were measured using standard standing position X-rays which were performed before surgery and at the final observation.

### Evaluation of clinical variables

Oswestry Disability Index (ODI), low back and leg pain based on visual analog scale (VAS) score, and the short form 36 health survey questionnaire (SF-36) were evaluated postoperatively and at the final observation.

### Complications

Neurological deterioration including the muscle weakness and numbness of the leg, and the other complication during surgery were assessed from inpatient records, outpatient visit records, operative records, and the radiographic images including MPR of CT. The intraoperative endplate injury of 2 mm over and the anterior longitudinal ligament injury were evaluated from the radiographic images including MPR of CT within 1 week after surgery. Proximal junctional kyphosis (PJK) and hardware failures such as cage subsidence according to the Marchi classification [22], screw breakage, and rod fracture were evaluated from the radiographic images including MPR of CT at the final observation. Finally, the causes of reoperation and its proportion were investigated.

### Statistical analyses

All t-tests were performed after confirming the normality of investigated data by the Shapiro-Wilk test. If the variables included in this study were not normally distributed, the Wilcoxon signed rank test was employed accordingly. Comparison of the required time to perform LIR at the fused vertebrae and XLIF at the other non-fused intervertebral spaces was conducted using the t-test. A paired t-test was used to evaluate the SLA and SCA at intervertebral segments treated with LIR pre-operatively and at 1-year after surgery. SLA 1-year after surgery was compared with the lordotic angle of inserted cage using a paired t-test. Spinopelvic parameters and global spinal alignment were compared between using pre-operative and final post-operative measurements using a paired t-test. ODI, VAS, and SF-36 were compared using a paired t-test or the Wilcoxon signed rank test. All statistical analyses were performed using JMP data analysis software, version 14 (SAS Institute Inc., Cary, NC, USA). *P*-values < 0.05 were considered statistically significant.

## Results

### Patient demographic, clinical, and surgical data

All patients in the study were females, with mean age 70.3 ± 4.8 years (range, 62–78 years), and BMI 24.4 ± 2.1 (mean ± standard deviation). Mean T-score was − 1.2 ± 0.8 and all patients used injection of teriparatide 2 months or more prior to surgery. Mean follow-up period was 28.4 ± 15.3 months. There were 2 fused vertebrae operated by LIR at L1/2, 6 at L2/3, 7 at L3/4, and 6 at L4/5 (Table 1). FR was 58.5 ± 22.9 (range, 17–100). Long fusion surgery from the thoracic to the pelvis was conducted in 13 cases and short fusion surgery within the lumbar spine was performed in 4 cases.

**Table 1:** Demographic, clinical, and surgical data

Case	Age	T-score	UIV	LIV	Follow-up periods (years)
1	71	−2.4	L2	L5	5
2	75	− 0.8	T9	S2	5
3	70	−1.8	L1	L5	5
4	70	−1.2	T9	S2	2
5	78	−1.4	L1	L5	2
6	68	−1.3	L1	L5	2
7	71	−1.4	T9	S2	2
8	74	0	T9	S2	2
9	78	−1.3	T9	S2	2
10	73	−2.4	T9	S2	2
11	67	−1	T10	S2	2
12	67	−1.2	T3	S2	2
13	70	−0.4	T9	S2	2
14	63	−1.7	T6	S2	2
15	67	−1.5	T8	S2	1
16	78	−1.7	T10	S2	1
17	62	0.5	T9	S2	1

*UIV* upper instrumented vertebra, *LIV* lower instrumented vertebra

### Radiographic and clinical outcomes in LIR segments

The average time for LIR was 21.3 ± 9.7 min and was not significantly different from that for XLIF (18.6 ± 6.8 min). The estimated blood loss per single segment during anterior surgery was 38.7 ± 53.2 mL. The total time to perform anterior surgery was 189.6 ± 48.4 min. The mean blood loss during anterior surgery was 134.1 ± 172.8 mL and the mean blood loss per single segment during anterior surgery was 38.7 ± 53.2 mL. Concerning invasive side, 14 levels were accessed from the convex side (non-fused side) and 7 levels were from the concave side (fused side). Regarding bone union pattern, total numbers of Proietti classification type II and III which mean bone bridges formation only inside internal spaces of the cage was 5 cases. Total numbers of type IV, V, VI, and VII which mean bone bridges formation inside internal spaces of the cage and outside the cage was 16 cases (76.2%) (Table 2). Bone union rate was 100.0%.

**Table 2:** Clinical outcomes associated with intervertebral levels treated with LIR

Case	Treated levels with LIR	Preoperative fusion ratio of fused vertebrae (a/b in Fig. 2)	Time to treat fused vertebrae (min)	Lordotic angles of XLIF cage (A)	Segmental lordotic angles 1-year after surgery (B)	(B)/(A)*100 (%)	Intraoperative endplate injury	Cage subsidence according to Marchi classification^22^	Bone union pattern according to Proietti classification^17^ 1-year after surgery
1	L4/5	52.2	–	10	8	80	+	I	VI
2	L4/5	37.0	–	15	15	100	–	I	VI
3	L4/5	48.2	–	10	9	90	–	I	II
4	L3/4	17.2	20	10	16	160	–	0	IV
5	L2/3	75.6	20	15	14	93	–	0	VI
6	L4/5	91.5	36	15	16	107	–	0	VI
7	L1/2	78.9	14	10	9	90	–	II	VI
8	L3/4	18.3	11	15	10	67	–	0	IV
	L4/5	35.0	13	15	12	80	–	0	IV
9	L3/4	100.0	20	15	19	127	–	II	VI
10	L3/4	52.5	–	15	11	73	–	I	II
11	L2/3	61.4	28	15	17	113	–	I	III
	L3/4	69.5	16	15	14	93	–	0	VI
12	L3/4	88.7	36	15	17	113	+	III	V
	L4/5	51.6	17	15	11	73	–	0	IV
13	L3/4	79.7	45	15	14	93	–	0	IV
14	L2/3	30.9	14	15	8	53	+	I	IV
15	L2/3	58.5	17	15	19	127	–	0	III
16	L2/3	57.9	25	10	12	120	–	I	III
17	L1/2	70.0	17	10	6	60	+	I	VI
	L2/3	53.2	13	10	8	80	–	0	VI

*LIR* lateral interbody release, *XLIF* extreme lateral interbody fusion

Concerning the segmental alignment, the SLA improved significantly from − 7.6 ± 9.2 degrees preoperatively to 7.0 ± 8.8 degrees at the final observation and the SCA improved significantly from 19.1 ± 7.8 degrees preoperatively to 8.7 ± 5.9 degrees at the final observation (*P* < 0.0001, and < 0.0001, respectively). The intra-observer variability for the evaluations of SLA before surgery and 1-year after surgery, and SCA before surgery and 1-year after surgery were 0.99, 0.98, 0.99, and 0.98, respectively. The interobserver variability was 0.90, 0.84, 0.94, and 0.92, respectively.

### Radiographic and clinical results in the whole spine

Table 3 summarizes the radiological evaluations of spinopelvic parameters pre- and postoperatively. There were significant differences in LL, PI minus LL, PT, SS, SVA between before surgery and at the final observation. Regarding parameters of coronal alignment, there were also significant differences in the Cobb angle and C7-CSVL between before surgery and at the final observation.

**Table 3:** The comparison of sagittal and coronal spinal parameters between before surgery and at the final observation in patients with minimum 2-years follow-up

Variables	Before surgery (*N* = 14)	At the final observation (*N* = 14)	*P* value
PI (deg.)	55.7 ± 12.3	–	–
PT (deg.)	41.1 ± 8.5	30.8 ± 8.1	< 0.0001**
SS (deg.)	14.6 ± 12.1	24.9 ± 8.0	< 0.0001
LL (deg.)	3.1 ± 21.6	38.6 ± 13.1	< 0.0001
PI-LL (deg.)	52.6 ± 18.1	17.1 ± 15.5	< 0.0001
SVA (mm)	134.3 ± 45.9	56.8 ± 44.8	< 0.0001
Cobb angle (deg.)	34.7 ± 9.0	13.3 ± 8.2	< 0.0001
C7-CSVL (mm)	28.3 ± 21.3	15.9 ± 13.8	0.012

*PI* pelvic incidence, *PT* pelvic tilt, *SS* sacral slope, *LL* lumbar lordosis, *SVA* sagittal vertical axis, *C7-CSVL* the absolute values of postoperative deviation of C7 plumb line off central sacral vertical line, mean ± standard deviation, a paired *t* test was used after the normality of the data was examined using the Shapiro-Wilk test

Table 4 summarizes the comparison of clinical outcomes between before surgery and at the final observation in minimum 2-years follow-up patients. The mean ODI, mean VAS score for low back pain, and the mean VAS score for leg pain improved between before surgery and at the final observation significantly. There were significant differences in each domain of SF-36 except social functioning.

**Table 4:** The comparison of clinical outcomes between before surgery and at the final observation in patients with minimum 2-years follow-up

Variables	Before surgery (*N* = 14)	At the final observation (*N* = 14)	*P* value*
ODI	50.9 ± 13.1	23.4 ± 19.6	< 0.0001
VAS			
Low back pain (mm)	68.7 ± 24.1	20.3 ± 26.6	< 0.0001
Leg pain (mm)	59.4 ± 32.5	27.1 ± 27.3	0.017
SF-36			
Physical functioning	33.2 ± 17.7	61.4 ± 21.1	0.0001
Role physical	22.9 ± 34.5	58.2 ± 30.1	< 0.01**
Bodily pain	26.9 ± 11.5	54.4 ± 26.5	0.002
General health	43.6 ± 16.1	54.1 ± 18.6	< 0.05**
Vitality	35.9 ± 18.4	57.9 ± 16.4	0.002
Social functioning	57.1 ± 29.7	70.5 ± 28.4	> 0.1**
Role emotional	25.0 ± 31.7	69.6 ± 31.6	< 0.01**
Mental health	45.9 ± 23.6	62.2 ± 19.7	0.02

*ODI* Oswestry Disability Index, *VAS* Visual analog scale score, *SF-36* the short form 36 health survey questionnaire, mean ± standard deviation, *P-value t-test for dependent samples, **Due to deviations from the normal distribution by the Shapiro-Wilk test, the Wilcoxon signed rank test was used

### Complications

There were no instances in which LIR was abandoned intraoperatively. Perioperative complications included one case of temporary iliopsoas muscle weakness and 3 cases of temporary leg pain or numbness. No injury of the anterior longitudinal ligament was observed. There was one case of an intravertebral osteotomy at wrong site. Intraoperative endplate injury occurred in 4 cases. Incidence of severe cage subsidence following LIR was 14.3% at 1 year after surgery (Table 2). No cage breakage was observed. In one patient, rod breakage occurred 6 months after surgery. The patient did not have to undergo reoperation because she was asymptomatic. There was one instance of screw breakage of S2 alar iliac fixation in patient with fragile unstable fracture of the pelvis in addition to ASD, requiring reoperation to more rigid fusion construct to the pelvis using double alar iliac screws. There was one patient requiring partial implant removal surgery because of skin trouble related to PJK. The overall cumulative reoperation rate was 11.8%. There were no reoperations associated with failure of the segment that underwent LIR.

### Case presentation

**Fig. 3:**
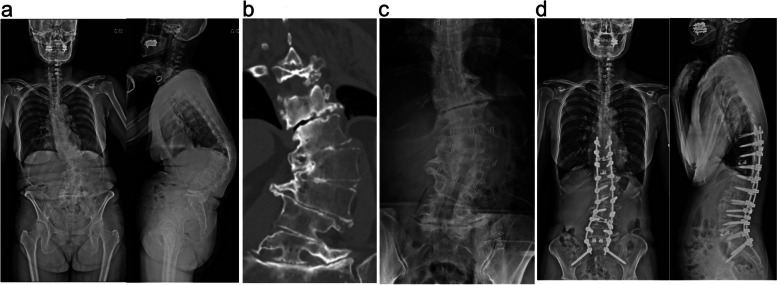
**a** Preoperative frontal and lateral view radiographs of a 62-year-old woman with low back pain demonstrated severe kyphosis and coronal spinal malalignment. **b** Multiple fused vertebrae at L1–2, L2–3, and L4–5 were found in multiplanar reconstruction of computed tomography. **c** The frontal radiograph exhibited LIR at L1/2 and L2/3 and XLIF at L3/4 were subseuqently performed. **d** Radiographs 1-year after surgery demonstrated that postoperative sagittal and coronal alignment were improved in this case. LIR; Lateral interbody release, XLIF; extreme lateral interbody fusion

A 62-year-old woman with low back pain due to ASD was indicated for combined anterior-posterior single-staged corrective fusion surgery (Fig. 3a). Auto-fused vertebrae were observed at L1/2, L2/3, and L4/5 on CT (Fig. 3b). Due to severe kyphosis in the upper lumbar spine and low PI (=30), the surgical plan was to perform LIR at L1/2 and L2/3 and LLIF at L3/4 and to avoid L4/5 XLIF placement in the anterior procedure (Fig. 3c). Blood loss and operative time during the anterior procedure were 160 mL and 144 min, respectively. Subsequently, posterior fusion with pedicle screw insertion, posterior release, and PLIF at L5/S1 and T11/12 level were performed in the prone position. Standing radiographs 1 year after surgery demonstrated improved spinal alignment parameters (Fig. 3d) and improvement in her preoperative symptoms.

## Discussion

Multi-segment interbody fusions are increasingly being utilized for the correction of adult spinal deformity, and the lateral approach is a common modality for these procedures. Multilevel LLIF for ASD has demonstrated less invasiveness compared to traditional three column osteotomies, significant radiographic corrective capabilities, and high fusion rate [4, 13–20]. Many patients with more advanced degenerative changes have autofusion of the anterior column across the endplates, more typically along the concavity of severe curves. Traditionally, vertebral autofusion would be surgically managed by three column osteotomies, which result in greater perioperative morbidity. Our group developed and performed lateral interbody release in these patients.

This is the first study, to our knowledge, to characterize the clinical and longer-term radiographic outcomes in patients who underwent anterior column realignment via lateral access interbody release to fused vertebrae, in the context of ASD correction. We found that LIR for the fused vertebrae, with remnant disc space on MRI, was feasible using the typical instruments for LLIF, regardless of the degree of preoperative fusion ratio. Our study demonstrated improvements in clinical and radiologic parameters at 2-year follow-up in LIR patients, and there were no instances of nonunion at LIR segments at final follow up. Therefore, the LIR technique may be a useful procedure when addressing fused vertebrae in the context of ASD correction.

Additionally, we characterized the intraoperative parameters and perioperative complications for LIR and found them to be similar to internal controls (non-fused segments) as well as external comparative data. In terms of blood loss, Oliveira et al. described 23.4 mL per level in patients who underwent stand-alone XLIF, which is comparable to the 38.7 mL per level osteotomy demonstrated in our study [23]. Subsidence rates following LIR were similar to those described in traditional LLIF procedures, perhaps due to endplate and osteophyte sclerosis in these patients, perhaps conferring increased biomechanical stability despite changes from the osteotomy [22, 24]. Lateral approaches in general carry a higher risk profile of lower extremity symptoms, specifically the incidence of psoas weakness, ranging from 13.6 to 30.8%, and sensory changes, ranging from 3.1 to 60.7% [25]. This LIR cohort had an 11.8% incidence of psoas weakness and 17.6% incidence of thigh numbness, which are within the range of previously reported data for traditional LLIF procedures. In terms of instrumentation failures, we found a 5.9% incidence of rod fracture after LIR, comparable to previously demonstrated rod fracture rate of 7.5% after corrective surgery using LLIF with posterior fusion for ASD [26]. In summary, LIR is a safe and effective technique for correction of ASD with anterior vertebral autofusion, with complication profiles similar to those demonstrated with traditional LLIF in ASD correction.

This study has several limitations, many of which are inherent in a case series of a novel procedural intervention. Namely, this study involves a limited patient population, largely resultant from the smaller incidence of vertebral autofusion compared to more typical ASD pathoanatomy. Further, this series serves as a feasibility study and proof of concept of this novel surgical strategy, as we wanted to critically evaluate this technique before broadening its application. Additionally, this study was performed at a single institution and affiliated surgeons, limiting generalizability of our results as they might apply when performed by a broader group of surgeons. Another limitation in terms of application would be that we included only a specific pattern of anterior vertebral autofusion as they occur in ASD patients, with some remnant disc on MRI, making these results less generalizable to other patterns of vertebral autofusion. Despite these acknowledged limitations, we feel this study provides a methodologically sound analysis of a novel procedural strategy.

## Conclusions

This is the first study, to our knowledge, to characterize the clinical and longer-term radiographic outcomes in patients who underwent anterior column realignment via lateral access interbody release of fused vertebrae, in the context of ASD correction. Clinical and radiographic outcomes at 2-year follow-up were significantly improved from preoperative metrics. Intraoperative and perioperative complication profiles for LIR were comparable to those demonstrated in traditional LLIF. We believe that the LIR technique for anterior column correction is a safe and effective strategy when indicated during ASD surgery.

## Data Availability

All data generated or analyzed during this study are included in this published article.

## Abbreviations

LIR: Lateral interbody release
DLS: Degenerative lumbar scoliosis
SLA: Segmental lordotic angle
SCA: Segmental coronal angle
PI: Pelvic incidence
LL: Lumbar lordosis
XLIF: Extreme lateral interbody fusion
ODI: Oswestry Disability Index
VAS: Visual analog scale
SF-36: Short form 36 health survey questionnaire
ASD: Adult spinal deformity
PSO: Pedicle subtraction osteotomy
VCR: Vertebral column resection
LLIF: Lateral lumbar interbody fusion
CT: Computed tomography
MRI: Magnetic resonance imaging
PLIF: Posterior lumbar interbody fusion
BMI: Body mass index
FR: Fusion ratio
MPR: Multiplanar reconstruction
PT: Pelvic tilt
SS: Sacral slope
SVA: Sagittal vertical axis
Cobb angle: The angle measured between the superior endplate of the most tilted vertebra cranially and the inferior endplate of the most tilted vertebra caudally
C7-CSVL: Deviation of the C7 plumb line from the central sacral vertical line
PJK: Proximal junctional kyphosis
